# Importance of Zinc Homeostasis for Normal Cardiac Rhythm

**DOI:** 10.2174/011573403X299868240904120621

**Published:** 2024-09-19

**Authors:** Pejman Kokhabi, Reza Mollazadeh, Seyedeh Fatemeh Hejazi, Aida Hossein Nezhad, Hamidreza Pazoki-Toroudi

**Affiliations:** 1 School of Advanced Medical Sciences, Tehran Medical Branch, Islamic Azad University, Tehran, Iran;; 2 Department of Cardiology, School of Medicine, Imam Khomeini Hospital Complex, Tehran University of Medical Sciences, Tehran, Iran;; 3 School of Advanced Medical Sciences, Tonekabon Medical Branch, Islamic Azad University, Tonekabon, Iran;; 4 Department of Physiology, Physiology Research Center, Faculty of Medicine, Iran University of Medical Sciences, Tehran, Iran

**Keywords:** Zinc, arrhythmia, trace elements, heavy metal, ion channels, ATP levels

## Abstract

Current arrhythmia therapies such as ion channel blockers, catheter ablation, or implantable cardioverter defibrillators have limitations and side effects, and given the proarrhythmic risk associated with conventional, ion channel-targeted anti-arrhythmic drug therapies, a new approach to arrhythmias may be warranted. Measuring and adjusting the level of specific ions that impact heart rhythm can be a simple and low-complication strategy for preventing or treating specific arrhythmias. In addition, new medicines targeting these ions may effectively treat arrhythmias. Numerous studies have shown that intracellular and extracellular zinc concentrations impact the heart's electrical activity. Zinc has been observed to affect cardiac rhythm through a range of mechanisms. These mechanisms encompass the modulation of sodium, calcium, and potassium ion channels, as well as the influence on beta-adrenergic receptors and the enzyme adenylate cyclase. Moreover, zinc can either counteract or induce oxidative stress, hinder calmodulin or the enzyme Ca (^2+^)/calmodulin-dependent protein kinase II (CaMKII), regulate cellular ATP levels, affect the processes of aging and autophagy, influence calcium ryanodine receptors, and control cellular inflammation. Additionally, zinc has been implicated in the modulation of circadian rhythm. In all the aforementioned cases, the effect of zinc on heart rhythm is largely influenced by its intracellular and extracellular concentrations. Optimal zinc levels are essential for maintaining a normal heart rhythm, while imbalances-whether deficiencies or excesses-can disrupt electrical activity and contribute to arrhythmias

## 1 INTRODUCTION

Cardiovascular diseases (CVDs) are a leading cause of global mortality, contributing significantly to disability [1]. In 2019, CVDs accounted for approximately 17.9 million deaths worldwide, representing 32% of all global fatalities [2]. Among these conditions, arrhythmias are a significant concern, with their incidence and prevalence increasing with age [3]. Arrhythmias are closely linked to heightened risks of sudden cardiac death, heart failure, and stroke, leading to reduced quality of life, increased mortality, and substantial healthcare costs [4].

Arrhythmias are predicted to affect 1.5% to 5% of the general population, with atrial fibrillation being the most prevalent [5]. A study in the United Kingdom involving over 500,000 individuals revealed that 2.35% exhibited abnormal baseline heart rhythms [6]. Current treatment options for arrhythmias include ion channel blockers, catheter ablation, and cardioverter defibrillator implantation. However, these interventions are associated with significant adverse effects and limitations. For instance, ion channel blockers can have proarrhythmic effects, while catheter ablation carries risks such as pericardial effusion and stroke [7-9]. Therefore, addressing the underlying factors contributing to arrhythmias, such as disturbances in ionic homeostasis, could serve as a more effective and cost-efficient approach to the prevention and treatment of Arrhythmias.

While the connections between ions like sodium, potassium, magnesium, and calcium with arrhythmias are well-established [10], the role of zinc is less commonly assessed. Recent findings suggest that zinc, a potential biomarker for cardiovascular health, can influence heart function and rhythm through various mechanisms [11]. Zinc modulates the activity of critical ion channels, including those selective for sodium, potassium, and calcium, which are essential for proper electrical impulse conduction and contractility within the myocardium [12-16]. Additionally, zinc regulates excitation-contraction coupling in cardiomyocytes by influencing calcium dynamics, a process fundamental to heart function [17]. This trace element also substituted for zinc also contributes to cellular energy production by participating in ATP synthesis [8, 18].

Mechanistically, zinc mitigates the stimulatory effects of the sympathetic nervous system on the heart by inhibiting adenylyl cyclase activity, thereby reducing cyclic adenosine monophosphate (cAMP) levels [19]. This helps counteract the positive chronotropic and inotropic effects mediated by the sympathetic nervous system. Furthermore, zinc shows potential in arrhythmia control by inhibiting calmodulin, a calcium-binding protein involved in various cellular signaling pathways [20]. Moreover, Zinc's antioxidant properties offer protection against oxidative stress, a significant contributor to arrhythmogenesis [21, 22]. Its involvement in mitophagy, the selective degradation of dysfunctional mitochondria, helps regulate ATP and reactive oxygen species (ROS) levels, potentially hindering arrhythmia progression [23].

Furthermore, this element prevents aging-related cardiac dysfunction by specifically targeting mitochondria and regulating the pro-inflammatory response [22, 24-26].

However, maintaining zinc homeostasis is critical, as an imbalance can be detrimental. High intracellular free zinc levels can reduce cellular energy production and increase ROS (reactive oxygen species) production, contributing to the development of arrhythmias [27, 28]. Elevated zinc concentrations may also disrupt voltage-dependent potassium currents, leading to altered cardiac function [24]. Thus, preserving normal zinc levels is essential for maintaining normal cardiac rhythm and function, highlighting zinc's potential as a therapeutic target in arrhythmia management.

Given zinc's multifaceted role in cardiac health, understanding the mechanisms by which zinc influences heart function is paramount. This manuscript reviews the current knowledge on zinc homeostasis and its impact on cardiac rhythm, highlighting the potential therapeutic implications for preventing and managing arrhythmias.

## 2 ZINC HOMEOSTASIS

Zinc, a crucial trace element, is found throughout the body with a total content of approximately 2-3 grams [29, 30]. Interestingly, nearly all (99.99%) of this zinc resides within cells [31]. Muscle and bone tissues hold the largest reserves, accounting for 60% and 30% of total body zinc, respectively, followed by the liver at 5% [31].

Zinc is primarily bound to proteins in the bloodstream, with 80% bound to albumin and 20% to α2-macroglobulin [32]. The concentration of free Zn^2+^ ions in plasma is low, ranging from 0.1 to 1.0 nanomolar [33].

Healthy adults typically maintain serum zinc levels within a reference range of approximately 70 to 120 µg/dL or 10.71 to 18.36 µmol/L [34]. Deviations from this range can indicate underlying health issues. This highlights the importance of efficient homeostatic mechanisms, as the body tightly regulates zinc levels despite variations in dietary intake [31]. To maintain this balance, the recommended daily intake (RDI) of zinc for adults is around 15 mg [34].

### 2.1 Systemic Zinc Homeostasis

Maintaining zinc homeostasis in humans primarily occurs within the digestive system and involves two key mechanisms: regulating zinc absorption from the intestine and its excretion in feces through pancreatic secretions [35]. The absorption of zinc predominantly occurs in the small intestine, specifically in the duodenum and proximal jejunum, facilitated by the Zip4 transporter [34].

In cases of high zinc intake, passive diffusion or attachment to the enterocyte apical membrane occurs, aided by metallothionein and other cysteine-rich proteins [36]. Enterocytes can store zinc as metallothionein or transport it across the basolateral membrane using the ZnT1 transporter [37]. After entering the bloodstream, zinc is transported to the liver, where it is either stored or bound to proteins such as albumin and released back into circulation. Zinc is transported from circulation to various tissues, including muscle and bone [37]. In the pancreas, ZIP5 facilitates the uptake of plasma zinc, which is then incorporated into zymogen granules by ZnT2 and subsequently excreted through pancreatic secretions [35]. Zinc can also be eliminated *via* gastrointestinal epithelial cell shedding and excretion [38]. In addition, the kidneys regulate zinc elimination, resulting in a daily urinary loss of approximately 500 to 800 µg zinc [39].

### 2.2 Cellular Zinc Homeostasis

The normal intracellular labile zinc level ([Zn^2+^]i) in cardiomyocytes is between5 pM and 1 nM under physiological conditions [29].

The main factors contributing to cellular zinc homeostasis are zinc transporter proteins, zinc-binding molecules like metallothionein, zinc sensors like metal-responsive transcription factor (MTF)-1, and Transient Receptor Potential Melastatin 7 channels TRPM7 [39]. Zinc transporter proteins (ZIP and ZnT) act as gatekeepers: ZIP (Zinc/Iron-regulated transporter-like proteins) transport zinc into the cytoplasm from extracellular fluid or intracellular vesicles, while ZnT (zinc transporter proteins) transport zinc out of the cell or into intracellular vesicles [40]. Metallothioneins are vital in buffering and storing zinc by redistributing Zn^2+^ ions to apo-proteins and maintaining appropriate labile Zn^2+^ concentrations. They regulate intracellular zinc by selectively binding it when levels become potentially harmful and then releasing it in a controlled manner [41]. Under conditions of zinc overload, additional metallothionein is produced to bind the excess zinc for excretion [42]. Metal-responsive transcription factor (MTF)-1 is a crucial regulator for MTs and ZnT1 genes, detecting intracellular zinc levels [43]. It binds to metal-responsive elements in promoters, upregulating transcription of target genes like ZnT-1 and metallothionein, allowing cells to export or buffer high intracellular zinc concentrations [43]. Finally, the ion channel TRPM7 plays a crucial role in regulating cellular zinc levels by facilitating Zn^2+^ currents. It also conducts important cations like Mg^2+^ and Ca^2+^ and toxic metals such as Ni^2+^ and Cd^2+^. Suppression of TRPM7 reduces intracellular Zn^2+^ availability. Reactive oxygen species (ROS) trigger TRPM7 to release Zn^2+^ from M7Vs intracellular vesicles and reduce cellular damage. However, increased zinc influx through TRPM7 may lead to neuronal cell death in conditions like ischemia, creating a debate about the protective *versus* harmful effects of TRPM7-mediated zinc influx [44].

Zinc is stored within cells in three major forms.: 1. Zinc ions (Zn^2+^) bound to proteins, such as metallothionein (MT), which is non-exchangeable and non-reactive zinc and represents around 54% of the zinc pool; 2. Zinc ions (Zn^2+^) localized within organelles and vesicles or bound to ligands, which form an exchangeable and reactive zinc pool (44.7%. of zinc pool); and 3. Free zinc ions dispersed throughout the cytoplasm, representing only 1% of the zinc pool [45].

**Fig. (1):**
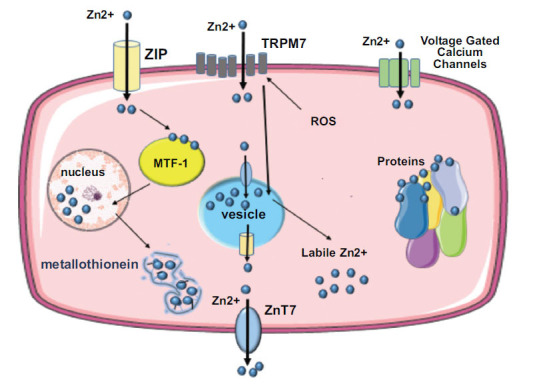
Zinc enters cardiac cells *via* ZIP transporters, TRPM7 channels, and voltage-dependent calcium channels. It is predominantly stored in intracellular vesicles, organelles, metallothionein, and specific ligands. ROS can induce TRPM7-dependent release of Zn^2+^ from M7Vs vesicles into the cytoplasm. Elevated zinc levels within the cell trigger enhanced activation of the transcription factor MTF-1, leading to increased production of metallothionein and enhanced buffering capacity to maintain optimal zinc levels within the cell. **Abbreviations:** MTF-1: Metal-Responsive Transcription Factor 1, ROS: Reactive Oxygen Species, TRPM7: Transient Receptor Potential Melastatin 7.

**Fig. (2):**
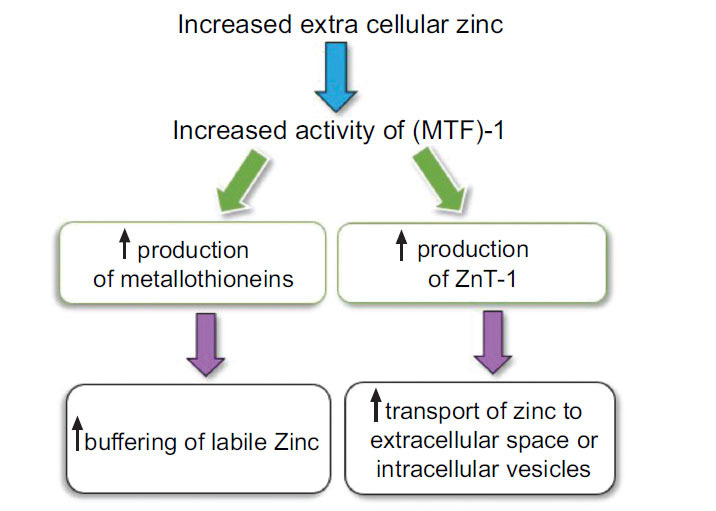
Illustrates that when extracellular zinc levels rise, it triggers the activation of Metal-responsive transcription factor (MTF)-1. Consequently, MTF-1 promotes the synthesis of metallothioneins, enhancing the capacity for zinc buffering. Additionally, MTF-1 stimulates the production of the ZnT-1 transporter, facilitating the transportation of zinc to intracellular vesicles or the extracellular space.

However, this tightly regulated pool can be significantly perturbed under certain pathological conditions. Acute exposure to oxidants, for instance, can trigger a 30-fold increase in labile Zn^2+^ concentration, while persistent hyperglycemia can lead to a 2-fold elevation [36]. Notably, elevated cytosolic labile Zn^2+^ levels have been implicated in alterations of cardiomyocyte excitability, ionic conductance, and arrhythmogenesis (Figs. **1** and **2**) [46].

### 2.3 Epidemiology and Etiology of Zinc Deficiency and Zinc Toxicity

The body has limited zinc reserves, necessitating replenishment through dietary intake. An estimated two billion people globally are at risk of health issues due to inadequate zinc levels. Current data indicates that about 17.3% of the global population consumes zinc-deficient diets, putting them at risk of deficiency [47]. Certain populations, such as older adults, are particularly vulnerable, with adults aged 75 and older at higher risk [48].

The primary cause of zinc deficiency in humans is insufficient dietary intake. However, other factors can also contribute, including excessive zinc losses through gastrointestinal conditions or urinary excretion. Conditions like chronic diarrhea, kidney disease, liver cirrhosis, infections, burns, excessive sweating, and the use of hemodialysis can lead to significant zinc loss [29, 49, 50]. Malabsorption of zinc, common in adolescents with inflammatory bowel disease, cystic fibrosis, celiac disease, or short bowel syndrome, can also cause deficient levels due to intestinal damage or disease [49].

Additionally, certain dietary substances can inhibit zinc absorption in the human digestive system when consumed excessively. These include phytates in whole grains and compounds such as copper, iron, calcium, and alcohol. This inhibition further exacerbates the risk of zinc deficiency in individuals with low intake or increased losses [51].

Zinc toxicity in humans, also known as hyperzincemia, primarily arises from excessive zinc intake through supplements and multivitamins, leading to acute or chronic health issues. Industrial exposure, particularly the inhalation of zinc oxide fumes during welding, smelting, and metal processing, is a significant cause, often resulting in “metal fume fever.” [52]. Chronic high-dose zinc supplementation can disrupt copper absorption, resulting in copper deficiency and neurological issues. Other symptoms of zinc toxicity include gastrointestinal distress, nausea, and vomiting [18, 52, 53].

## 3 IMPACT OF ZINC ON CARDIAC RHYTHM THROUGH ION CHANNELS

Zinc ions in the extracellular environment can positively and negatively affect the functioning of various ion channels [12]. This is because many proteins, particularly ion channels, contain specific residues such as histidine, cysteine, aspartate, and glutamate with a high zinc affinity. By interacting with these sites on both the extracellular and intracellular surfaces, zinc can alter the channel's structure, either activating or inhibiting its function [13]. Maintaining precise intracellular zinc levels is crucial for proper cardiac function and electrophysiology, as zinc's effects on these channels can profoundly influence heart rhythm and stability [29]. In normal conditions, intracellular zinc levels are tightly regulated. These regulated extracellular and intracellular zinc levels are essential for the proper function of excitable cells. When zinc concentrations deviate from their normal ranges, they can disrupt the cells' electrical properties, specifically altering their voltage-dependent responses [14].

### 3.1 Effect of Zinc on Cardiac Sodium Channels

Cardiac sodium channels (Nav1.5) are responsible for the rapid influx of sodium ions during the initial depolarization phase of the cardiac action potential, regulating sodium ion flow in the heart. Recent research has shown that zinc can bind to a site within or near the saxitoxin binding pocket on these channels, giving them an affinity for external zinc approximately 100 times greater than that of other sodium channel types [16]. This binding exerts a repulsive effect on sodium ion binding, similar to the way calcium and sodium ions repel each other when binding to structurally related calcium channels. It is proposed that zinc attaches directly to cardiac sodium channels, inducing a sub-conductance state where channel activity is reduced [16, 54]. A study investigates the effects of group IIb cations (cadmium, zinc, mercury) on the cardiac fast sodium current (INa) in calf Purkinje fibers and guinea-pig ventricular cells. It finds that submillimolar concentrations of zinc and mercury significantly reduce INa, with mercury being the most potent. This reduction is voltage-independent and affects the inactivation kinetics of sodium channels, causing a shift in voltage dependence at higher concentrations. The results indicate that zinc, like other group IIb cations, modulates cardiac sodium channels by binding to them and partially inhibiting their sodium ion conduction (Table **1**) [54].

### 3.2 Effect of Zinc on Cardiac Potassium Channels

Cardiac potassium channels are essential for maintaining heart rhythm by regulating the repolarization of cardiomyocytes, shaping the action potential duration, and controlling the resting membrane potential [13]. As the most functionally diverse ion channels, they contribute to various phases of the cardiac action potential, ensuring proper electrical activity. Dysfunction in these channels can lead to arrhythmias such as long QT syndrome, atrial fibrillation, and ventricular tachycardia [55]. The various types of K+ channels have overlapping roles, resulting in some functional redundancy [56].

Zinc exerts a complex and diverse influence on cardiac potassium channels. It has been shown to cause a varied depolarizing shift of the ionic current half-activation potential and significantly decrease the activation kinetics of most K+ channels [15].

#### 3.2.1 Effect of Zinc on Voltage-gated Potassium Channels

##### 3.2.1.1 Effect of Zinc on Kv11.1 (Human Ether-a-go-go Channel or hERG)

This channel is responsible for IKr (rapid delayed rectifier current), which is crucial for late phase 3 repolarization. Zinc reduces the ion current of the human Kv11.1 channel by binding to its histidine residues, potentially leading to prolonged action potential duration and delayed repolarization [57].

##### 3.2.1.2 Effect of Zinc on Kv1.5

The Kv1.5 channel is responsible for generating IKur (ultra-rapid delayed rectifier current), which plays a significant role in the repolarization of atrial cells. Zinc has an inhibitory effect on this channel, which can result in an extended atrial action potential duration. This prolongation may increase the vulnerability to atrial fibrillation [15].

##### 3.2.1.3 The Effect of Zinc on Kv1.4 and other Voltage-gated Potassium Channels

Kv1.4 contributes to Ito (transient outward current) and is involved in early repolarization (phase 1). Zinc inhibits this channel by binding to their extracellular regions or histidine residues. This inhibition generally leads to prolonged repolarization and altered action potential duration [13]. The activation kinetics of Kv10.2 and Kv12.1 channels are decelerated when zinc ions bind to the extracellular aqueous cleft of these channels [57]. In contrast, the Kv1.2 channels are insensitive to zinc ions [13].

#### 3.2.2 Effect of Zinc on Large-conductance BK Channels

These contribute to repolarization, particularly under conditions of increased intracellular calcium. Zinc activates BK channels through interactions with their intracellular domains. This activation can enhance repolarization, potentially shortening action potential duration and affecting cellular excitability [13].

#### 3.2.3 Effect of Zinc on TREK-1 and TASK-3 Channels

TREK-1 channels in the heart are stretch-sensitive potassium channels that play a crucial role in cardiac electrophysiology. They maintain the resting membrane potential, influence the duration of action potentials, and participate in the heart's mechano-electrical coupling mechanism. These channels activate in response to mechanical stress, and any disruption in their normal function may lead to stretch-induced cardiac arrhythmias [58]. They have attracted significant interest as potential therapeutic targets for treating atrial fibrillation [59]. Zinc exerts an inhibitory effect on both channel types with different potency. According to a study, the concentration of zinc required to achieve half-maximal inhibition (IC_50_) of TASK-3 channels is approximately 12.7 ± 1.0 μM, whereas for TREK-1 channels, a much higher concentration of 659 ± 94 μM is needed [60].

#### 3.2.4 Effect of Zinc on ATP-sensitive K+ Channels (KATP)

KATP channels link cellular metabolism to membrane excitability. They are typically closed under normal conditions but open during metabolic stress. Zinc activates these channels, which can enhance repolarization and hyperpolarize the membrane potential [13]. This activation could be cardioprotective during ischemia by reducing cellular excitability and energy demand [13].

The overall effect of zinc on cardiac action potential depends on the balance of its inhibitory and activating effects on different channel types. Generally, the inhibition of voltage-gated K+ channels tends to prolong the action potential, while activation of BK and KATP channels can shorten it [61]. The net result may vary depending on the specific cardiac region, zinc concentration, and pathophysiological conditions. This complex interplay underscores the importance of maintaining precise zinc homeostasis for normal cardiac electrophysiology [13].

Table **2** summarizes the effect of zinc on different cardiac potassium channels.

### 3.3 Effect of Zinc on Cardiac Calcium Channels

Cardiac calcium channels, primarily L-type and T-type, are crucial for shaping the cardiac action potential, particularly during the plateau phase. They play a key role in excitation-contraction coupling by triggering calcium-induced calcium release from sarcoplasmic reticulum. Additionally, these channels contribute to pacemaker activity in nodal cells, helping to generate and maintain normal cardiac rhythm [62].

Zinc functions as a negative regulator for all calcium channels [62], including various types of high-voltage-activated calcium channels like L-type and T-type channels, even in the presence of sufficient Ca^2+^ levels either directly at the membrane level or intracellularly through second-messenger systems in a voltage-dependent manner [14, 29, 63]. Evidence indicates that intracellular Zn2+ can effectively inhibit the L-type calcium current (ICaL), with a half-maximal inhibitory concentration identified at 12.7 nM [63].

While zinc cannot freely cross the cell membrane, certain calcium-permeable channels, like gated calcium and Transient Receptor Potential (TRP) channels, allow zinc to permeate with electrical stimulation [18]. This ability of zinc to enter the cell through specific channels, such as ligand-gated and voltage-activated calcium channels, leads to alterations in their behavior [24]. Increased intracellular labile zinc (Zn^2+^ >1 nM) can inhibit Cardiac L-type Calcium Channels (LTCCs), which is crucial for calcium influx during the plateau phase (phase 2) of the cardiac action potential. This inhibition reduces calcium entry into the cell, which can decrease the subsequent release of calcium from the sarcoplasmic reticulum [14, 54]. Increased intracellular zinc can also interfere with calcium-induced calcium release mechanisms by inhibiting the ryanodine receptors (RyRs) on the sarcoplasmic reticulum. Inhibition of RyRs can reduce the amount of calcium released into the cytoplasm, affecting muscle contraction and other calcium-dependent processes [64].

Inhibition of L-type calcium channels (LTCCs) and modulation of ryanodine receptors (RyRs) by zinc can reduce cardiac contractility and alter cardiac rhythms, potentially contributing to conditions such as arrhythmias and heart failure. This mechanism is evident in several pathological conditions, including aldosteronism, where intracellular zinc homeostasis is disturbed by uncontrolled increases in intracellular labile zinc (over 200% in aldosteronism) [63, 65].

When cardiomyocytes are in pathological conditions, elevations in both extracellular and intracellular zinc can affect the ICa (L-type calcium current), although not in the same manner [14]. A study found that extracellular zinc at a concentration of 10 µM leads to a 30% reduction in L-type Ca^2+^ channel currents (ICa) in ventricular myocytes without altering the current-voltage relationship. Intracellular zinc, when increased from normal levels to approximately 7 nM using Zn-pyrithione, causes significant inhibition of ICa and shifts the voltage dependency of L-type Ca^2+^ channels [14]. The findings indicate that pathological conditions in cardiomyocytes can alter calcium entry by augmenting extracellular and intracellular zinc levels.

In another study using isolated ventricular cardiomyocytes from rats and mice, exposure to 32 μM zinc (Zn^2+^) demonstrably elevated intracellular zinc levels to approximately 13 nM within 3-5 minutes [65]. This increased intracellular Zn^2+^ concentration modulated calcium dynamics by inhibiting Ca^2+^ influx through LTCCs, leading to decreased intracellular Ca^2+^ concentrations during both systole and diastole, along with a diminished SR Ca^2+^ load. These alterations in calcium handling ultimately manifested as suppressed systolic function, with reduced peak sarcomere shortening, and enhanced diastolic relaxation, with increased diastolic sarcomere length [65].

Another investigation examined the effect of extracellular 32 μM zinc acetate (Zn^2+^) application on the function of isolated cardiomyocytes from hyperglycemic (HG) rats [66]. The study found that zinc enhances the relaxation and pacing frequencies of cardiomyocytes in HG by likely reducing intracellular calcium overload through several mechanisms including: enhanced removal of cytosolic Ca^2+^ *via* Sarcoplasmic/Endoplasmic Reticulum Calcium ATPase 2a (SERCA2a), enhanced calcium removal *via* the Na^+^/Ca^2+^ Exchanger (NCX), reduced Ca^2+^ influx *via* L-type channels, decreased Ca^2+^ leak through ryanodine receptors and limiting RyR phosphorylation. Thus, the presence of extracellular Zn^2+^ was associated with a reduction in contraction force and the normalization of incomplete relaxation in conditions of hyperglycemia. As a result, exposure to extracellular zinc improved cardiomyocyte relaxation in high-glucose (HG) conditions, helping to counteract the harmful effects of hyperglycemia on calcium regulation. Zinc reduced calcium overload and improved relaxation, especially during higher pacing frequencies, indicating its potential therapeutic benefit for treating diabetic cardiomyopathy by enhancing cardiac relaxation function [66]. Overall, the results of these studies indicate that normal levels of intracellular Zn^2+^ play a crucial role in regulating calcium release and contribute to the normalization of calcium handling. At low physiological concentrations, Zn^2+^ can enhance RyR2 activity, leading to increased calcium release from the sarcoplasmic reticulum (SR) and increased muscle contraction. On the other hand, in pathophysiological states, elevated intracellular Zn^2+^ levels (Zn^2+^ > 1 nM) inhibit LTCC activity, reduce calcium influx, and may increase abnormal calcium release *via* RyR2, contributing to diastolic calcium leak, impaired contractility, and arrhythmias [63-65].

#### 3.3.1 Effect of Zinc on Cardiac Calcium Channels through its Effect on ZnT Transporters

Zinc may influence calcium channels by upregulating the expression of Zinc Transporter 1 (ZnT-1). ZnT-1 has been identified as an endogenous modulator of LTCC function [67]. The inhibition of LTCC by ZnT-1 appears to occur through its interaction with the LTCC beta-subunit, leading to a reduced trafficking of the LTCC alpha1-subunit to the cell surface [67]. A study on rats demonstrated that increased ZnT-1 expression inhibits L-type calcium channels, shortens the atrial effective refractory period (ERP), and causes dispersion in action potential repolarization [67].

Table **2** summarizes the effect of zinc on different cardiac calcium channels.

### 3.4 Effect of Zinc on Acid-sensing Ion Channels

Acid-sensing ion channels (ASICs) are proton-activated channels that primarily transport sodium ions, with ASIC1a also capable of transporting calcium [68]. Six ASIC isoforms exist, forming various channel configurations. ASICs are widely distributed in the nervous system and cardiac tissue, playing crucial roles in sensory transduction, including mechanical, chemical, and pain signals [69].

In the cardiovascular system, ASICs are involved in baroreceptor and chemoreceptor functions. ASIC2 isoform is key in baroreceptor mechanosensation, contributing to blood pressure regulation through the arterial baroreflex [68]. ASIC3 isoform is believed to be the primary chemosensor in glomus cells, responding to changes in pH, oxygen, and carbon dioxide levels [70, 71].

During myocardial ischemia, extracellular pH decreases and afferent innervation becomes overexcited. As a result, ASICs have been proposed as molecular sensors for cardiac ischemic pain [72]. Specifically, ASIC2a/3 and ASIC3 isoforms are thought to be responsible for sensing ischemic cardiac pain. In contrast, ASIC1a plays a significant role in the injury caused by ischemia rather than in pain sensation [68].

Depending on the ASIC subtype, zinc can either stimulate or inhibit ASICs by binding to specific sites on these channels [73]. Zinc has been shown to inhibit ASIC1b at a half-maximal inhibitory concentration of 37 μM [74]. Since ASIC1b is involved in ischemia-reperfusion injury and the resulting arrhythmia, maintaining proper intracellular or extracellular zinc levels might help prevent arrhythmias caused by reperfusion injury [75].

Studies have shown that Zn(^2+^) stimulates channels containing homomeric and heteromeric ASIC2a subunits, including ASIC2a, ASIC1a/2a, and ASIC2a/3 [76]. Zinc also has a stimulatory effect on ASIC1a/3 channels at concentrations above 250 μM. Moreover, zinc pretreatment inhibits ASIC3 in a concentration-dependent manner, blocking both the peak and sustained functions of these channels [77].

Given the role of ASICs in the autonomic innervation of the heart and their involvement in baroreceptor and chemoreceptor signal transduction, zinc's impact on these channels could potentially prevent or induce arrhythmias by influencing autonomic nerves. However, the current understanding of this topic is limited, requiring further research.

## 4 IMPACT OF ZINC ON CARDIAC RHYTHM THROUGH ITS EFFECT ON INTRACELLULAR PH

**Fig. (3):**
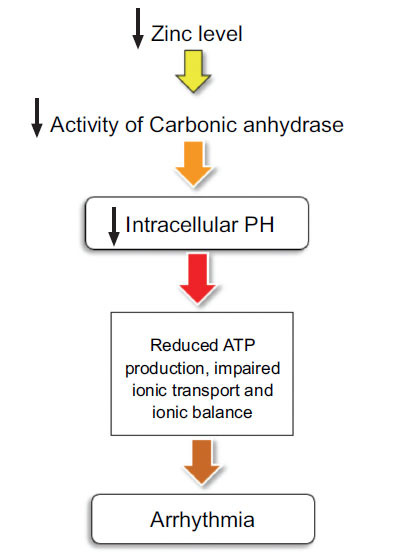
Zinc deficiency can result in reduced activity of the carbonic anhydrase enzyme, leading to a decrease in intracellular pH, an imbalance of ions, and the development of cardiac arrhythmia.

Carbonic anhydrase is a metalloenzyme that regulates the acid-base balance by catalyzing the reversible conversion of carbon dioxide and water into bicarbonate and hydrogen ions. This enzyme binds to the Na+/H+ Exchanger and enhances its activity [78]. Zinc is an essential cofactor for carbonic anhydrase [79], and its deficiency can reduce the enzyme's activity, resulting in an intracellular pH imbalance [79]. Unregulated intracellular pH can cause a decrease in the release of calcium ions from the sarcoplasmic reticulum, leading to reduced contractility of the cardiac muscle and inhibition of the flow of calcium ions. Additionally, decreased intracellular pH can result in decreased ATP production, impaired ion transport, and the potential development of arrhythmias [80]. Acidosis has been shown to significantly prolong AV node delay and partial or complete AV node block and bradyarrhythmias [81]. However, the direct effect of zinc on intracellular PH requires additional investigations (Fig. **3**).

## 5 IMPACT OF ZINC ON CARDIAC RHYTHM THROUGH ATP PRODUCTION

Both zinc deficiency and zinc overload can negatively impact the production of adenosine triphosphate (ATP) by interfering with mitochondrial enzymes, inducing oxidative stress, and damage to mitochondria [18, 27, 82].

Several mitochondrial enzymes and electron transport chain subunits rely on zinc as a cofactor [82]. It has been demonstrated that when Zn^2+^ is chelated, mitochondrial complexes become inactive, halting ATP production [18].

On the other hand, various conditions, such as ischemia-reperfusion injury, have the potential to cause an increase in intracellular free Zn^2+^ concentrations [83]. This elevation in Zn^2+^ levels can initiate oxidative stress, resulting in the induction of mitochondrial permeability transition, loss of mitochondrial membrane potential, impaired mitochondrial respiration, and decreased ATP production [27, 83]. It has been proposed that increasing intracellular zinc to submicromolar levels can also inhibit glycolysis by inhibiting Glyceraldehyde-3-phosphate dehydrogenase (GAPDH) and phosphofructokinase, as well as the Krebs cycle by inhibiting the α-ketoglutarate dehydrogenase complex [27]. Moreover, this zinc concentration can inhibit the bc1 complex of mitochondrial cytochrome, resulting in decreased ATP production [84]. ATP plays a critical role in maintaining ion balance in cardiac cells. Myosin ATPase uses a significant portion of ATP in contractile filaments, while the sarcolemmal Na+/K+ ATPase, and the sarcoplasmic reticulum's Ca^2+^ ATPase also utilize ATP added [85]. Insufficient ATP levels have substantial implications for cellular function, including the impairment of calcium reuptake into the sarcoplasmic reticulum, leading to elevated cytosolic calcium levels [86]. Furthermore, the activity of Na+/K+ ATPases, essential for maintaining ion gradients and membrane potential, is hampered. As a result, there is an imbalance in intracellular ion distribution and a decrease in contractility, leading to an environment favorable to the development of arrhythmias [86].

## 6 EFFECT OF Zn^2+^ ON ADENYLATE CYCLASE AND β-ADERNERGIC STIMULATION

Zn^2+^ has been shown to block adenylyl cyclase, specifically the AC5 and AC6 isoforms found in cardiomyocytes, which has been linked to the reduction of cAMP signaling. Therefore, at various concentrations of cellular Zn^2+^, cAMP-mediated signaling may be affected or enhanced [35].

In a study on rats, intracellular Zn^2+^ at even lower concentrations (<1 nM) significantly inhibited β-adrenergic stimulation by inhibiting the adenylyl cyclase [63].

It has been demonstrated that both basal intracellular and extracellular Zn^2+^ modulate transmembrane Ca^2+^ movements and their regulation by β-adrenergic stimulation [29].

## 7 EFFECT OF ZINC ON CALMODULIN AND CALCIUM/CALMODULIN-DEPENDENT PROTEIN KINASE II (CAMPK-II) ACTIVITY

Calcium, calmodulin, and calcium/calmodulin-dependent protein kinase II (CaMKII) are essential for cardiac function. Calmodulin undergoes conformational changes upon binding to calcium, which allows it to interact with and regulate various enzymes. One of these enzymes is CaMKII, which is activated by the calcium-calmodulin complex [87].

Once activated, CaMKII phosphorylates numerous Ca handling proteins including phospholamban (PLB), ryanodine receptors (RyR), and L-type calcium channels, which are crucial for transarcolemmal calcium influx (ICaL). Furthermore, CaMKII influences the activity of sarcolemmal sodium and potassium channels..activation of CaMKII, enhances calcium influx through L-type calcium channels and promotes calcium release from the sarcoplasmic reticulum [88], dysregulation or excessive activation of CaMKII has been implicated in several cardiac pathologies, including arrhythmias, contractile dysfunction, and patholog- ical remodeling. Consequently, CaMKII inhibitors have been proposed to prevent cardiac arrhythmias [88].

Zinc (Zn^2+^) exerts multiple regulatory effects on CaMKII. Research has shown that zinc can alter CaMKII to a state that prevents it from responding to calcium signals [20].

Previous studies proved that inhibiting CaMKII can effectively counteract the effects of adrenergic stimulation, thus resulting in an anti-arrhythmic impact. A notable study conducted on mice with a specific genetic mutation (RyR2(R4496C+/-)), which predisposes them to catecholamine-induced persistent ventricular tachyarrhythmia, found that administration of KN-93, a CaMKII inhibitor, effectively prevented the occurrence of arrhythmias. This study demonstrated that KN-93 reduced the susceptibility to ventricular tachyarrhythmia by stabilizing the RyR2 channels and preventing calcium leakage [89].

In another study involving rabbits with heart failure, KN-93 was shown to significantly reduce arrhythmia inducibility and slow the initiation of ventricular tachycardia. The researchers observed that CaMKII inhibition decreased spontaneous calcium release events, which are critical triggers for arrhythmias. These findings suggest that CaMKII inhibition may have broad anti-arrhythmic effects, particularly in the context of heart failure [90].

Further evidence supporting the therapeutic potential of CaMKII inhibitors comes from studies on oral CaMKII inhibitors. A demonstrated that RA608, an orally active CaMKII inhibitor, improved contractile function and prevented arrhythmias in a mouse model of heart failure. The researchers found that RA608 treatment significantly reduced arrhythmia incidence and improved overall cardiac function, highlighting the potential of CaMKII inhibitors as a therapeutic strategy for heart failure patients [91].

Additionally, studies on the role of zinc in regulating CaMKII have shown that zinc can modulate CaMKII activity and prevent its excessive activation. For instance, a study demonstrated that zinc can inhibit CaMKII activity by binding to its regulatory domain, thereby preventing it from responding to calcium signals [20]. This regulatory effect of zinc provides an additional layer of control over CaMKII activity and suggests potential therapeutic applications in conditions characterized by CaMKII dysregulation.

In conclusion, experimental studies underscore the critical role of CaMKII in cardiac function and its potential as a therapeutic target. The inhibition of CaMKII through pharmacological agents such as KN-93 and RA608 has shown promise in preventing arrhythmias and improving cardiac function in preclinical models. Moreover, the regulatory effects of zinc on CaMKII offer further insights into the complex regulation of this kinase and its potential for therapeutic modulation [89-91].

## 8 ZINC'S EFFECT ON CARDIAC RHYTHM *VIA* RYANODINE RECEPTORS

Ryanodine receptor 2 (RyR2) is a crucial transmembrane protein located in the sarcoplasmic reticulum (SR) of cardiomyocytes. It is vital in regulating calcium release from the SR, essential for muscle contraction and excitation-contraction coupling (ECC) [92]. Calcium release through RyR2 from the SR is tightly regulated, with intermittent release during systole and cessation during diastole. Dysregulated calcium release during diastole has been associated with the development of arrhythmias in genetic disorders such as catecholaminergic polymorphic ventricular tachycardia (CPVT) and acquired cardiac diseases like atrial fibrillation and heart failure. Recent insights into ECC have identified SR Ca^2+^ release through RyR2 as a key mechanism in the initiation and persistence of both atrial and ventricular arrhythmias [93, 94].

RyR2 stability can be compromised by inherited mutations, such as those seen in CPVT, or acquired modifications, including oxidation, nitrosylation, and phosphorylation. These alterations can lead to abnormal Ca^2+^ release during diastole, triggering arrhythmias [93].

In heart failure (HF) and CPVT, dysfunctional RyR2 can cause abnormal spontaneous diastolic Ca^2+^ leaks from the SR, contributing to the formation of delayed afterdepolarizations, which are believed to underlie fatal arrhythmias. Research indicates that targeting RyR2 may be an effective anti-arrhythmic strategy for managing pathological Ca^2+^ release from the SR [92].

Research has demonstrated that zinc modulates both the frequency and amplitude of calcium waves in cardiomyocytes in a concentration-dependent manner. Physiological levels of Zn^2+^ can induce calcium release from the sarcoplasmic reticulum without the activation of cytosolic Ca^2+^, revealing a new role for intracellular Zn^2+^ in modulating RyR2 gating and influencing calcium dynamics in cardiomyocytes [64].

A study showed that under physiological conditions, free Zn^2+^ ≤ 1 nM concentrations potentiated RyR2 activity, but activating levels of cytosolic Ca^2+^ were required for channel activation. At concentrations of free Zn^2+^ > 1 nM, Zn^2+^ became the main activating ligand for RyR2, eliminating the need for Ca^2+^ to activate the channel. Under these conditions, channel gating was altered, and calcium waves persisted even when intracellular calcium levels were reduced to sub-activating concentrations as long as Zn^2+^ remained at 1 nM [64].

In other words, under conditions of zinc dyshomeostasis (intracellular Zn^2+^ > 1 nM), RyR2 channels may become abnormally active or “leaky,” potentially leading to irregular calcium release during diastole. This mechanism could contribute to the progression of heart failure and fatal arrhythmias [64]. These data underscore that maintaining zinc homeostasis is critical for normal cardiac rhythm due to its significant impact on RyR2-mediated calcium release.

## 9 ZINC, CARDIAC RHYTHM, AND OXIDATIVE STRESS

Oxidative stress, resulting from an imbalance between reactive oxygen/nitrogen species and antioxidants, can damage cellular components and is associated with arrhythmias such as atrial fibrillation and ventricular arrhythmias [22, 25]. Elevated reactive oxygen species (ROS) disrupt ion channel function, leading to imbalances in sodium, potassium, and calcium concentrations and altered membrane permeability [86, 95]. These changes contribute to cardiac electrical instability, prolonged action potential duration, and cellular calcium overload, all promoting arrhythmias [96, 97]. Furthermore, increased ROS levels affect various aspects of cardiac function, including mitochondrial activity, gap junction remodeling, ATP production, ryanodine receptor 2 function, sarcoplasmic reticulum calcium release, and CaMKII activation. These multifaceted effects of oxidative stress on cardiac physiology collectively contribute to the development of different types of arrhythmias [22, 25, 86, 95, 98, 99].

Emerging evidence suggests that targeting reactive oxygen species (ROS) release pathways in mitochondria using antioxidants could have promising anti-arrhythmic effects by reducing cardiac oxidation [86]. Both *in vitro* and *in vivo* research have shown that zinc exerts antioxidant effects and protects cells from oxidative injury [100]. However, it should be noted that both zinc deficiency and excess can lead to oxidative stress and cytotoxicity [43]. The antioxidant effect of zinc is attributed to its ability to bind to thiol groups, preventing their oxidation [101, 102]. Zinc plays a vital role as a cofactor for essential enzymes involved in the antioxidant defense system, including superoxide dismutase and catalase [100, 103, 104]. It also promotes the upregulation of glutathione (GSH) biosynthesis, which is critical in maintaining cellular redox balance [43].

Moreover, zinc inhibits NMDA receptors, which are involved in calcium transport, and zinc deficiency can activate these receptors, resulting in elevated intracellular calcium levels [100]. Zinc further exerts its antioxidant effect by inhibiting NADPH-oxidase, regulating the expression and transcription of nuclear factor erythroid 2-related factor 2 (Nrf2), and promoting the synthesis of metallothioneins (MTs) [105]. These findings highlight the potential of zinc as a novel therapeutic target for cardiac complications associated with oxidative stress Research findings suggest that zinc may counteract oxidative stress associated with ischemia-reperfusion and diabetes, partly through the enhancement of the zinc-binding protein metallothionein's capacity [65].

While zinc can act as an antioxidant at normal concentrations, excessive zinc levels under pathophysiological conditions can have adverse effects. In particular, elevated cytosolic labile zinc ions (Zn^2+^) have been found to induce arrhythmogenic action potentials in left ventricular cardiomyocytes by oxidizing protein thiols and depleting cellular ATP [46].

The accumulation of Zn^2+^ within mitochondria has negative consequences, including inhibiting α-ketoglutarate dehydrogenase and complexes I and III of the electron transport chain (ETC) [24, 27]. Consequently, this inhibition causes an elevation in reactive oxygen species (ROS) production. Higher concentrations of Zn^2+^ have also been correlated with augmented production of reactive oxygen/nitrogen species, thiol oxidation, and hyper-phosphorylation of intracellular proteins and kinases, all of which impact the contractile mechanism of cardiomyocytes [24].

## 10 ZINC, MITOPHAGY, AND CARDIAC RHYTHM

Mitochondrial dysfunction disrupts intracellular ion balance and membrane excitability due to decreased ATP production and excessive generation of reactive oxygen species (ROS), increasing susceptibility to cardiac arrhythmias [106]. Mitophagy, a specialized form of autophagy, protects the heart by selectively targeting and degrading damaged or defective mitochondria, reducing ROS production, and guarding against various cardiac disorders [107, 108].

Maintaining mitochondrial homeostasis and eliminating toxic substances through mitophagy is crucial for inhibiting or slowing the progression of arrhythmias [23, 109]. Zinc has been shown to alleviate oxidative stress in mitochondria by promoting mitophagy [110]. It influences mitophagy through multiple mechanisms, including regulating key proteins in mitophagy initiation and controlling signaling pathways that govern this process [111].

One critical protein involved in initiating mitophagy is PTEN-induced putative kinase 1 (PINK1), which accumulates on the outer mitochondrial membrane (OMM) of damaged mitochondria [112]. Zinc promotes PINK1 stabilization on the OMM, leading to the activation of Parkin [113]. This activation triggers the relocation and initiation of Tank Binding Kinase 1 (TBK1) towards impaired mitochondria, activating autophagy receptors and forming autophagosomes around the damaged mitochondria [114].

Autophagy receptor proteins contain a zinc finger (ZNF) domain that binds to ubiquitinated proteins [115]. Studies have demonstrated that zinc treatment enhances mitophagy through PINK1 and Beclin 1 *via* ERK, thereby preventing mitochondrial ROS generation under hypoxia/reoxygenation conditions [110].

Furthermore, zinc stimulates autophagy by activating the AMPK/mTOR signaling pathway [116]. It also regulates mitophagy by modulating the small ubiquitin-like modifier (SUMO) system, which is involved in various cellular processes [117]. Additionally, zinc may influence autophagy by promoting gene expression by activating Metal-responsive transcription factor 1 (MTF1) and microRNA synthesis [111].

These findings highlight the critical role of zinc in maintaining mitochondrial function and protecting against cardiac arrhythmias by promoting mitophagy and regulating key signaling pathways.

### 10.1 Effect of Zinc on Mitophagy through its Effect on the Ubiquitin-proteasome System (UPS)

Zinc-fingers (ZNFs) are proteins that rely on zinc as a structural cofactor to maintain their conformation and have a wide variety of molecular functions, including DNA recognition, activation of transcription, regulation of apoptosis, signal transduction, protein folding or degradation, and ubiquitin-mediated protein degradation [118]. In mitophagy, ubiquitin is attached to the PINK1 fragment on the surface of mitochondria to mark them for degradation by the ubiquitin-proteasome system (UPS) and autophagy machinery [119]. The effect of zinc on the ubiquitin-proteasome system is complex and can be both inhibitory and stimulatory depending on the context [120]; however, ZFAND5/ZNF216, a protein that contains zinc finger domains, is an activator of the 26S proteasome, which stimulates overall protein degradation by the ubiquitin-proteasome pathway [121].

## 11 ZINC, AGING, AND CARDIAC RHYTHM

The aging process is a major risk factor for cardiac dysfunction [24].

The heart, during aging, exhibits a depressed mechanical activity due to mitochondria-originated increases in ROS and decreased antioxidant capacity [24]. This process can lead to dysregulation in Ca^2+^ homeostasis, increased sarcoplasmic reticulum Ca^2+^-leak through RyR2 channels (which can cause structural remodeling), electrical instability, action potential prolongation, advanced fibrosis, increased rate of mitochondrial defects and oxidative stress pressure in cardiomyocytes [24, 122-124]. A study on aged rats found that their cardiac rhythm and action potentials were characterized by increased spontaneous action potentials, indicating a higher propensity for arrhythmias. The ventricular cardiomyocytes exhibited impaired intracellular Ca^2+^ handling and altered K+-channel currents, contributing to instability in cardiac electrical activity [125]. This suggests that aging affects the electrophysiological properties of the heart, making it more susceptible to rhythm disturbances [125]. Treatment of these rats with MitoTEMPO, a mitochondria-targeting antioxidant, significantly improved mitochondrial structure and function, reduced mitochondrial reactive oxygen species (ROS) production, protected against spontaneous action potentials, and improved K+-channel currents and intracellular Ca^2+^ handling [125]. A study suggests that zinc deficiency in the heart can accelerate cardiac aging and lead to increased oxidative stress, heart failure, ischemia-reperfusion injury, and other cardiovascular diseases [24]. On the other hand, an overload of zinc in the mitochondria contributes to oxidative stress and cellular senescence, which can be major factors in the progression of cardiovascular diseases and heart aging [24]. Therefore, targeting mitochondrial Zn^2+^ transporters pharmacologically to normalize zinc levels or using direct mitochondria-targeting antioxidant treatments could be effective strategies for preventing or treating cardiovascular dysfunction associated with aging [24].

## 12 ZINC, INFLAMMATION, AND CARDIAC RHYTHM

Research has shown a connection between inflammation and the development of arrhythmias, especially atrial fibrillation (AF). This link is due to mechanisms like oxidative stress, apoptosis, and fibrosis, which promote the formation of substrates for AF [126, 127]. For example, individuals with sepsis or critical illness have a higher likelihood of new-onset AF, and there is evidence connecting pneumococcal pneumonia to AF [128]. Biomarkers such as C-reactive protein (CRP) and cytokines also appear to predict the development and outcomes of cardiac arrhythmias [129].

Zinc plays a role in modulating the inflammatory process by targeting the Nuclear Factor Kappa B (NF-κB), a major regulator of pro-inflammatory responses and oxidative stress [130-132]. Oxidative stress can enhance the activation of NF-κB, a redox-sensitive transcription factor. NF-κB, in turn, can suppress the transcription of cardiac Na+ channels, leading to cardiac electrical remodeling and contributing to atrial fibrillation (AF) [133]. By reducing inflammatory cytokines, zinc helps control chronic inflammation [132]. Studies have shown that zinc supplementation increases the expression of A20, a zinc transcription factor that inhibits NF-κB activation, thereby reducing inflammatory cytokine production [22, 25, 26].

Another pathway zinc affects is the Tumor Necrosis Factor α (TNFα) signaling pathway. TNFα's biological effects are mediated through TNF receptor type-1 (TNFR1) and type-2 (TNFR2), which are expressed in various cardiac cells [134]. TNFα may contribute to AF by activating the TGF-β/Smad2/3 signaling pathway, inducing atrial fibrosis [135]. The molecular pathophysiology of AF involves electrical, structural, and contractile remodeling of the atrium, all facilitated by TNFα signaling [134]. TNFα also inhibits the production of sarcoplasmic reticulum Ca^2+^-ATPase in cardiomyocytes, promoting arrhythmia [128].

A double-blind, placebo-controlled study found that zinc treatment in the elderly increased plasma zinc levels and decreased plasma concentrations of CRP, TNFα, and IL-6 [132]. An inverse relationship between IL-6, TNFα, CRP, and serum zinc levels has been observed in 40-year-old adults [136]. High-sensitivity CRP is associated with AF development and persistence [137]. Zinc deficiency has been noted in patients with elevated oxidative stress and inflammatory status [132].

The NLRP3/caspase-1 inflammasome pathway is another inflammatory pathway involved in arrhythmias like AF [133]. This multiprotein complex regulates innate immunity and inflammatory signaling [138]. Activation of the NLRP3 inflammasome is crucial for the development of malignant ventricular arrhythmia post-myocardial infarction [139]. Zinc supplementation has been found to decrease NLRP3 and caspase-1 gene expression compared to placebo [140]. Prolonged zinc deficiency can impair lysosomal structural integrity, leading to increased activation of the NLRP3 inflammasome [141]. Thus, zinc, as an anti-inflammatory agent, may help prevent arrhythmias triggered by inflammation.

In light of the above, zinc's anti-inflammatory properties may help prevent arrhythmias associated with conditions like COVID-19 [142-144]. These multifaceted effects suggest zinc's potential as a preventive and therapeutic agent for inflammation-induced arrhythmias, warranting further research into its clinical applications.

### 12.1 Effect of Zinc on Inflammation through its Effect on Metallothionein

Metallothionein, a protein whose production relies heavily on the presence of zinc, exerts significant anti-inflammatory and immune-modulatory effects [145]. Furthermore, metallothioneins possess antioxidant properties, which further aid in mitigating inflammation [146, 147].

## 13 EFFECT OF ZINC ON CARDIAC RHYTHM THROUGH ITS EFFECT ON CIRCADIAN RHYTHM

Studies of 24-hour ECG recordings from healthy volunteers revealed that the normal cardiac rhythm follows a circadian rhythm that includes nocturnal bradycardia, increases in RR, PR, and QT intervals, and QRS length at night. This suggests slower AV node conduction, His-Purkinje conduction, and ventricular repolarization, respectively [147]. Certain life-threatening arrhythmias, such as ventricular tachycardia, are more likely to develop after awakening in the morning. This demonstrates that cardiac arrhythmias, like regular cardiac rhythms, follow a circadian pattern [148].

Two potential mechanisms can explain the effect of the circadian rhythm on cardiac function. One possibility is that the central circadian clock in the hypothalamus directly influences the heart's function by modulating the autonomic nervous system. This leads to increased parasympathetic activity at night and heightened sympathetic tone during the daytime. The second mechanism involves, a local circadian clock within the heart that drives 24-hour rhythms in the expression of ion channels. These fluctuations in ion channel expression can impact the occurrence of arrhythmias [148, 149].

Studies suggest that zinc is crucial in circadian rhythm modulation [150]. Photoreceptor cells, essential constituents of the circadian rhythm system, contain substantial quantities of zinc [151]. This high zinc concentration is critical for controlling the structure and function of rhodopsin, the light-absorbing pigment found in photoreceptors [151]. Beyond its role in these retinal cells, zinc is thought to participate in propagating nerve impulses between synapsed neurons within the circadian network. Additionally, zinc may modulate neuron excitability in brain parts integral to circadian regulation [150, 152]. Taken together, the considerable accumulation of zinc in key cells and structures indicates that it facilitates the proper timing of circadian cycles by influencing photoreceptor photopigments and neuronal signaling pathways. In addition several studies have found strong positive correlations between zinc and melatonin, a hormone that modulates circadian rhythms [153].

While several factors tightly regulate plasma zinc levels, these levels display a daily fluctuation, characterized by reduced concentrations in the evening and elevated concentrations in the morning [154].

**Fig. (4):**
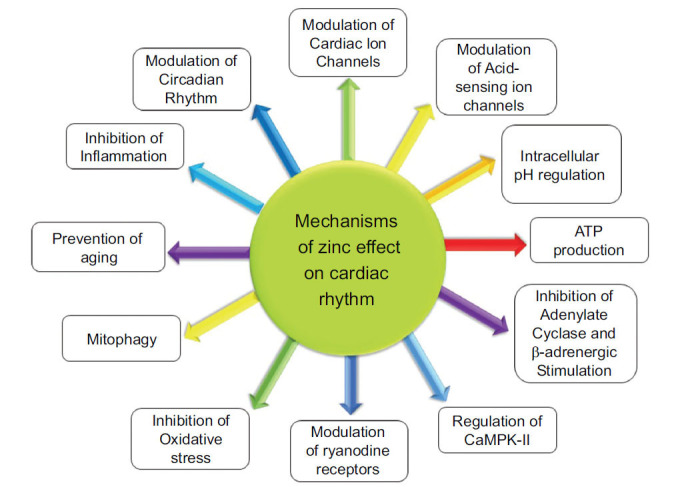
Summarizes the mechanisms by which zinc affects heart rhythm. Zinc plays an important role in several processes in the cardiovascular system, including its involvement in ion channel activity, membrane potential regulation, and signaling pathways that influence cardiac contraction and electrical conduction in the heart.

An experimental study showed that dietary zinc levels significantly influence circadian rhythms and lipid metabolism in male mice [155]. Zinc deficiency altered the expression of circadian clock genes Bmal1 and Per2, leading to a shift in their circadian rhythms (Fig. **4**) [155].

## CONCLUSION

Zinc, a trace element with diverse effects, influences various ion channels, including sodium, calcium, potassium, and acid-sensitive channels. It also exhibits antioxidant and anti-inflammatory properties, suggesting its potential role in preventing, controlling, or developing different arrhythmias through distinct mechanisms. Zinc impacts beta-adrenergic stimulation, cardiac ryanodine receptors, mitophagy, and the aging process in cardiac cells. Both zinc deficiency and excess can be detrimental to cells, leading to significant metabolic disturbances, particularly impaired excitation-contraction cycling in cardiomyocytes.

Therefore, maintaining normal serum and intracellular zinc levels, similar to those of sodium, potassium, and calcium ions, and assessing zinc concentrations in different arrhythmias seem to be crucial. Consequently, the use of medications or substances targeting cellular zinc transporters such as ZIP and ZnT transporters, TRPM7 channels, and transcription factors like Metal-responsive transcription factor (MTF)-1, shows promising potential as a therapeutic approach for treating cardiac dysfunction, including arrhythmias.

However, further research is necessary to fully comprehend the physiological mechanisms underlying zinc's anti-arrhythmic actions in cardiomyocytes. Advancing our understanding in this area will contribute to developing more effective approaches for arrhythmia treatment.

## Figures and Tables

**Table 1:** The effect of zinc on different cardiac sodium channels.

Major Channel Group	Subtype	Function in Cardiac Action Potential	Effect of Zinc	Effect of Inhibition on Action Potential
Voltage-Gated Sodium Channels (Nav) [16]	Nav1.5	Initiation and rapid depolarization of the cardiac action potential	Inhibition leads to reduced sodium influx	Decreased action potential amplitude and conduction velocity

**Table 2:** Effects of zinc on cardiac potassium channels.

Major Channel Group	Subtype (Other Names)	Function in Cardiac Action Potential	Effect of Zinc	Effect of Inhibition on Action Potential
*Voltage-Gated Potassium Channels (Kv)*	Kv11.1 (hERG, I_Kr) [56]	Repolarization phase, delayed rectifier K+ current (I_Kr)	Reduces ion current, inhibition	Prolonged repolarization, risk of arrhythmias
-	Kv10.2 [56]	Repolarization, delayed rectifier K+ current	Slows activation, inhibition	Prolonged repolarization, risk of arrhythmias
-	Kv12.1 [56]	Repolarization, delayed rectifier K+ current	Slows activation, inhibition	Prolonged repolarization, risk of arrhythmias
-	Kv1.4 (I_to) [13]	Early repolarization, transient outward K+ current (I_to)	Inhibition	Prolonged early repolarization, altered action potential
-	Kv1.5 (I_Kur) [15]	Repolarization in atria, ultra-rapid delayed rectifier K+ current (I_Kur)	Inhibition	Prolonged atrial action potential, risk of atrial arrhythmias
-	Kv1.2 [13]	Repolarization, delayed rectifier K+ current	No effect	N/A
*Inward Rectifier Potassium Channels (Kir)*	Kir2.1 (I_K1) [56]	Maintains resting membrane potential and late phase repolarization	No effect	N/A
Two-Pore Domain Potassium Channels (K2P)	TREK (KCNK2, K2P2.1) [59]	Modulates action potential duration and membrane excitability	Inhibition	Increased excitability, altered action potential
-	TASK (KCNK3, K2P3.1) [59]	Background K+ current regulates resting membrane potential	Inhibition	Increased excitability, altered resting potential
*Non-Voltage-Gated Potassium Channels*	KATP (pancreas/heart) [13]	Metabolic response, action potential shortening during stress	Activation/inhibition	Reduced protective shortening during stress
-	BK (Slo1, Maxi-K, BKCa) [13]	Repolarization, regulated by Ca^2+^ and voltage	Activation	Prolonged action potentials, altered excitability

**Table 3:** Effect of zinc on different cardiac calcium channels.

Major Channel Group	Subtype	Function in Cardiac Action Potential	Effect of Zinc	Effect of Inhibition on Action Potential
Voltage-Gated Calcium Channels	Cav1.2 (L-type) [13]	Plateau phase (phase 2), prolonged calcium influx	Inhibition	Reduced contractility, altered rhythm
-	Cav3.1 (T-type) [13]	Early depolarization, pacemaker activity	Inhibition	Reduced heart rate, altered pacemaker activity
Intracellular Calcium Release Channels	RyR2 [13]	Calcium release from the sarcoplasmic reticulum	Modulation (activation/inhibition)	Altered calcium release, impacting contractility
Transient Receptor Potential (TRP) Channels	TRPC3/6/7 [13]	Modulation of calcium influx, contributing to depolarization	Modulation (activation/inhibition)	Altered calcium homeostasis, affecting excitability and contractility

## LIST OF ABBREVIATIONS

AF: Atrial Fibrillation
ASICs: Acid-sensing Ion Channels
ATP: Adenosine Triphosphate
cAMP: Cyclic Adenosine Monophosphate
CPVT: Catecholaminergic Polymorphic Ventricular Tachycardia
CVDs: Cardiovascular Diseases
ERP: Effective Refractory Period
ETC: Electron Transport Chain
HG: Hyperglycemia
LTCCs: L-type Calcium Channels
MT: Metallothionein
MTF: Metal-responsive Transcription Factor
OMM: Outer Mitochondrial Membrane
RDI: Recommended Daily Intake
ROS: Reactive Oxygen Species
RyRs: Ryanodine Receptors
SR: Sarcoplasmic Reticulum
TNFα: Tumor Necrosis Factor α
TRP: Transient Receptor Potential
